# Clinical Trials in Turkey

**DOI:** 10.4274/Tjh.2013.0045

**Published:** 2013-06-05

**Authors:** Hilal İlbars

**Affiliations:** 1 Turkish Medicines and Medical Devices Agency Director of Clinical Trials Unit, Ankara, Turkey

**Keywords:** Clinical trial, Turkey

Turkey is an emerging economy in almost all sectors, and especially in pharmaceuticals. Turkey has had clinical trial legislation for a long time. There are also clinical trial regulations and guidelines. Turkey has taken major steps towards harmonizing its legislation with the European Union in the field of clinical research and currently Turkish regulations are totally in line with EC Directives (EC 2001/20 and EC 2005/28). The main objective of this article is to discuss the relevant parts of the minimum necessary information on current legislation related to clinical trials in Turkey, instead of detailing all current legislation related to the clinical trials in Turkey as of 2011. Details of legislation mentioned in the article can be found in the Official Gazette and on corporate websites. In order to protect the rights, well-being, and health of the subjects in accordance with internationally valid scientific and ethical standards, clinical trials must be performed within these legal regulation frameworks and attention should be paid to the presentation of quality and reliable data.

Turkey offers good opportunities with regard to the high number of enthusiastic physicians and, moreover, the higher availability of treatment-naive patients interested in participating in clinical trials.

Turkey is in the review phase to join the European Union and has undergone many important reforms involving patients rights, criminal law, specialized biotech drug dispensing acts, and so on. Along with policies and visions for scientific and technological development put forward by the EU in the mid-1990s, Turkey has taken major steps towards harmonizing with the EU in the field of clinical research and drug trials.

Turkey is known for its unique geographical location, straddling the continents of Europe and Asia with borders to 7 countries: Greece and Bulgaria to the northwest, Syria to the south, Iraq to the southeast, and Armenia, Georgia, and Iran to the east. It is a bridge to Europe from Asia and the Middle East.

Turkey is an emerging economy in almost all sectors, and especially in pharmaceuticals. Turkey has had clinical trial legislation for a long time. There are also clinical trial regulations and guidelines. Turkey has taken major steps towards harmonizing its legislation with the EU in the field of clinical research and, currently, Turkish regulations are totally in line with EC Directives (EC 2001/20 and EC 2005/28). Thus, there are ethics committees. According to the Turkish clinical trial legislation, after getting approval from an ethics committee, one needs to get permission for conducting clinical trials in Turkey from the Turkish Medicines and Medical Devices Agency (TMMDA). The TMMDA is working intensively to standardize ethics committee SOPs and applications.

Turkey has a population of 75 million people (median age: 30 years). Turkey also has well-reputed research sites with strong technical infrastructure capabilities at international standards.

The Clinical Trials Department of the TMMDA reviews clinical trials in Turkey. Under the Clinical Trials Department there are a phase evaluation unit, bioequivalence/ bioavailability evaluation unit, and a post-marketing surveillance evaluation unit.

The main objective of this article is to discuss the relevant parts of the minimum necessary information on current legislation related to clinical trials in Turkey, instead of detailing all current legislation related to the clinical trials in Turkey as of 2011. Details of legislation mentioned in the article can be found in the Official Gazette and on corporate websites.

The distribution of clinical drug trials conducted in accordance with legal regulation in Turkey between 01.01.1997 and 31.12.2012 is shown in Table 1.

Table 1 shows that there is a decrease in the number of clinical trials in 2010 and 2011; this is because of some legal problems about regulation of clinical trials in Turkey.

If we look the distribution of studies, the first 6 major areas are oncology, anesthesiology, the central nervous system, the cardiovascular system, endocrinology, and infectious diseases, followed by others.

When we analyze the number of studies related to hematology that were conducted in 2012, there were 6 Phase II studies, 18 Phase III studies, 1 Phase IV study, and 1 PMS study. In oncology these numbers were 6 Phase II and 16 Phase III studies.

Before planning to conduct any clinical trial on human subjects in Turkey, being informed of the following main legal regulations will be helpful.

In the Turkish Constitution of 1982, in the second part on fundamental rights and duties, article 17 of chapter 1, “Personal Inviolability, Material and Spiritual Entity of the Individual”, clearly states that clinical trials cannot be conducted without consent of the subject.

In Act No. 1219 on Medicine and Practicing of the Medical Profession, article 70 states that clinical trials cannot be conducted without consent, and if clinical trials on children or persons with legal disability are planned, obtaining consent of the legal guardian is obligatory.

Article 10 of Health Services Principal Act No. 3359 should also be known.

As mentioned in the second paragraph of “Experiments on Humans”, the 90th article of Act No. 5237 of the Turkish Penal Code, in order to rule out penal responsibility of a consent-based scientific experiment on a human being, it should be known by the subject, as mentioned in the first paragraph, that a “person conducting a scientific experiment on human beings will be sentenced to imprisonment for a term of from one to three years”.

If trials on children are planned, the 31/3/2005 – 5328/7 amended article of the Turkish Penal Code should be known. Accordingly, in order to reach the desired objective, scientific data obtained as a result of the research should necessitate conduct of the research on children; along with the consent of any child who is able to declare consent, the consent of parents or legal guardians must also be obtained; and authorized committees issuing approval for the research should involve pediatricians.

Especially if clinical trials involve special populations (such as disabled persons or children), the following articles of the Turkish Civil Code will be helpful to explain and obtain subjects’ informed consent: article 9-13 for legal capacity, article 14-16 for legal incapacity, article 23 for the protection of personality, article 404-410 for cases necessitating guardianship, article 411-412 for authority on guardianship procedures, article 413-416 for assignment of guardian, and article 417-418 for restraints of guardianship.

The 2008 revisions to the Declaration of Helsinki are essential.

Section V of the Act on Approval of Convention for the Protection of Human Rights and Dignity of the Human Being with regard to the Application of Biology and Medicine (“Oviedo Convention”): The Convention on Human Rights and Biomedicine is reserved specifically for scientific research and should be considered.

In assignments (especially for pharmacists), Act No. 657 on State Personnel and No. 6197 on Pharmacists and Pharmacies should be considered.

Articles 10 and 11 of the Medical Deontology Regulation highlight that it should be declared that the performed procedure is research and should be previously tested on experiment animals.

**Regulations**

1. Regulation on clinical trials issued on 13 April 2013 and numbered 28617 in the Official Gazette, should be known in all of its details.

2. In No. 28307 of the Official Gazette, dated 26 August 2011 and published on “Regulation on Promotion of Human Medicinal Products”, it is stated in section ğ of the 9th subparagraph of the 6th article that the license/ authorization holder will deliver human medicinal products, laboratory kits, and other such donations to be used in clinical trials directly to the responsible investigator. The 4th subparagraph of the 7th article clearly states that domestic and foreign investigator meetings of national and international multicenter clinical trials sponsored by the license/authorization holder will not be considered as congress or symposium participation, the nature of the meeting should be clearly stated in the submitted application, and it should be noted that the meeting is organized for this reason. Section g of the 1st subparagraph of the 9th article states that promotional samples are not allowed to be used as investigational products in clinical trials.

3. No. 21942 of the Official Gazette, dated 27 May 1994 and published on “Regulation for Evaluation of Bioavailability and Bioequivalence (BA/BE) of Pharmaceutical Products”, is especially important for the assessment of BA/BE studies.

4. For research with medical devices, No. 27957 of the Official Gazette, dated 7 June 2011 and published on “Regulation for Medical Devices” and “Regulation for Implantable Active Medical Devices”; No. 26398 of the Official Gazette, dated in 9 January 2007 and published on “Regulation for In Vitro Medical Diagnostic Devices”; and related TSE standards should be known.

5. Review of the sections of No. 23420 of the Official Gazette related to clinical trials, published 1 August 1998 on “Regulation for Patient’s Rights”, will be helpful.

There are also several guidelines that detail the regulations mentioned above.

**Guidelines for Operations:**

1. Good Clinical Practices Guidelines

2. Sections of Good Manufacturing Practices Guidelines Related to Investigational Products

3. Guidelines for Ethical Approach to Clinical Research Conducted on Pediatric Populations

4. Guidelines for Insurance Compensation for Clinical Trials

5. Guidance on the Collection, Verification, and Presentation of Adverse Reaction Reports Occurring During Clinical Trials

6. Guidelines Regarding Independent Data Monitoring Committees

7. In assigning field staff, “Regulation for Site Organization Management Principles in Clinical Drug Studies” should be considered

8. Guidelines for Archiving in Clinical Trials

9. “Guidelines for Storage and Distribution of Investigational Products Used in Clinical Trials”, prepared for guidance on issues of appropriate storage and distribution of investigational products used for clinical trials

10. Guidelines for Human Medicinal Products with Fixed Combinations

11. Guidelines for Biosimilar Medicinal Products

12. Guidelines for Principles and Essentials of Good Clinical Practices of Advanced Treatment Medicinal Products

13. Guidelines for Preparation of Audit Reports for Good Clinical Practices

14. Guidelines for Auditing of Good Clinical Practices of Laboratories Participating in Clinical Drug Trials

15. Guidance for Conduction of Good Clinical Practice Inspections of Sponsor and Contract Research Organization

16. Guidance for the Conduct of Good Clinical Practice Inspections to Bioanalytical Part, Pharmacokinetic and Statistical Analyses of Bioequivalence Trials

17. Guidelines for the Conduct of Auditing of Good Clinical Practices of Phase I Units

18. Guidelines for Observational Studies with Drugs

19. Prohibitions section of Guidelines for Off Label Drug Use should be considered in publications

20. “The Guidelines for Compassionate Use Program”, aiming to provide patients who have a severe or emergent, life-threatening disease, whose treatment with approved and available medicinal products in Turkey failed and who are not able to participate in clinical trials conducted clinical trials, with free-of-charge treatment for humanitarian reasons by the developer/supplier of drugs that are not registered in Turkey and either are or are not registered abroad.

**Guidelines for Submissions:**

1. Guidelines Regarding Application Format for Application to Ministry in Clinical Research

2. Guidelines Regarding Application Format for Application to Ethics Committee in Clinical Trials

SOP principles for ethics committees have also been published.

In accordance with the above-mentioned legal regulations, for certain studies approval of both an ethics committee and the Ministry of Health are required. These studies include:

• Clinical drug trials with drugs and drug compositions conducted on humans even if they are registered or authorized

• Clinical trials conducted with advanced treatment medicinal products

• Observational drug studies

• Clinical trials conducted with traditional herbal medicinal products

• Observational medical device studies

• Clinical trials conducted with medical devices

• Clinical trials conducted with cosmetic raw materials or products

• Clinical trials conducted with all other materials and products that may be tested on humans

• Bioavailability and bioequivalence studies

• Comparableness studies for biosimilar products

• Clinical trials with industrial advanced medicinal products

• Clinical trials with nonindustrial advanced medicinal products

• Stem-cell transplantation studies on human

• Organ and tissue transplantation studies

• New methods in surgical studies

• Gene therapy studies

In order to protect the rights, well-being, and health of subjects in accordance with internationally valid scientific and ethical standards, clinical trials must be performed within this legal regulation framework and attention should be paid to presenting quality and reliable data.

Turkey offers good opportunities with regard to the high number of enthusiastic physicians and, moreover, a higher availability of treatment-naive patients interested in participating in clinical trials.

## Figures and Tables

**Table 1:**
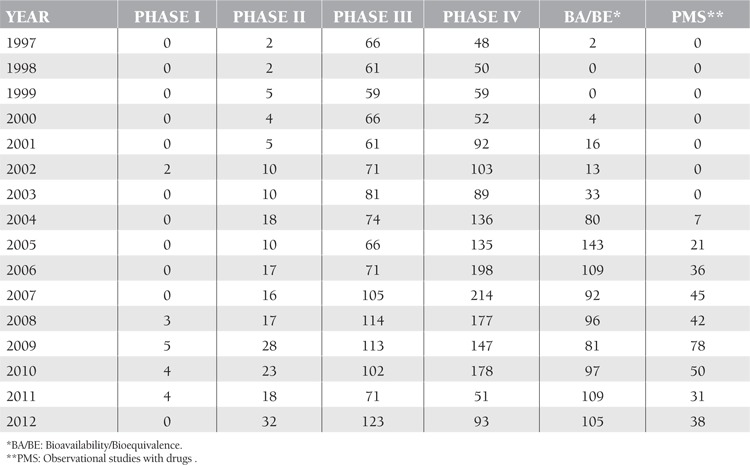
The distribution of clinical drug trials conducted in accordance with legal regulation in Turkey between 01.01.1997 and 31.12.2012.
